# Digital Phenotyping to Delineate Salinity Response in Safflower Genotypes

**DOI:** 10.3389/fpls.2021.662498

**Published:** 2021-06-16

**Authors:** Emily Thoday-Kennedy, Sameer Joshi, Hans D. Daetwyler, Matthew Hayden, David Hudson, German Spangenberg, Surya Kant

**Affiliations:** ^1^Agriculture Victoria, Grains Innovation Park, Horsham, VIC, Australia; ^2^Agriculture Victoria, AgriBio, Centre for AgriBioscience, Bundoora, VIC, Australia; ^3^School of Applied Systems Biology, La Trobe University, Bundoora, VIC, Australia; ^4^GO Resources Pty Ltd., Brunswick, VIC, Australia; ^5^Centre for Agricultural Innovation, School of Agriculture and Food, Faculty of Veterinary and Agricultural Sciences, The University of Melbourne, Melbourne, VIC, Australia

**Keywords:** high-throughput phenotyping, RGB imaging, salinity, salt tolerance, digital biomass

## Abstract

Salinity is a major contributing factor to the degradation of arable land, and reductions in crop growth and yield. To overcome these limitations, the breeding of crop varieties with improved salt tolerance is needed. This requires effective and high-throughput phenotyping to optimize germplasm enhancement. Safflower (*Carthamus tinctorius* L.), is an underappreciated but highly versatile oilseed crop, capable of growing in saline and arid environments. To develop an effective and rapid phenotyping protocol to differentiate salt responses in safflower genotypes, experiments were conducted in the automated imaging facility at Plant Phenomics Victoria, Horsham, focussing on digital phenotyping at early vegetative growth. The initial experiment, at 0, 125, 250, and 350 mM sodium chloride (NaCl), showed that 250 mM NaCl was optimum to differentiate salt sensitive and tolerant genotypes. Phenotyping of a diverse set of 200 safflower genotypes using the developed protocol defined four classes of salt tolerance or sensitivity, based on biomass and ion accumulation. Salt tolerance in safflower was dependent on the exclusion of Na^+^ from shoot tissue and the maintenance of K^+^ uptake. Salinity response identified in glasshouse experiments showed some consistency with the performance of representatively selected genotypes tested under sodic field conditions. Overall, our results suggest that digital phenotyping can be an effective high-throughput approach in identifying candidate genotypes for salt tolerance in safflower.

## Introduction

Salinity is one of the most severe abiotic constraints for crop production worldwide. Soil salinity can be due to primary causes, the inherent accumulation of sodium (Na^+^) from geological and meteorological process (dryland salinity), or develop as secondary salinity due to human settlement (transient or irrigation salinity; Rengasamy, 2002, 2006). Globally, over 900 million hectares or 6% of land are affected by saline or sodic soils (Rengasamy, 2002, 2006; Munns and Tester, 2008; Wicke et al., 2011). This is expected to expand to over 50% of arable land by 2050, due to climate change and mismanagement of irrigation, soil and land management practices (Pitman and Läuchli, 2002; Ivushkin et al., 2019). Salinity in agricultural areas causes a range of issues, including severe crop reductions and changes of soil biophysical properties (Rengasamy, 2010; McDonald et al., 2012).

Plants experience the effects of salt stress at all stages of development from germination to vegetative growth and reproduction, through complex biochemical and physiological interactions (Munns and James, 2003; Gengmao et al., 2015; Hussain and Al-Dakheel, 2018). Interactions can be shoot ion dependent, caused by ion toxicity and nutrient deficiency, or shoot ion independent, causing osmotic and oxidative stresses (Flowers, 2004; Munns and Tester, 2008; Roy et al., 2014). Crop salt tolerance is therefore considerably variable between species, as well as between genotypes and cultivars of the same species, due to reliance on different salt tolerance components (Janardhan et al., 1986; Maas, 1993; La Bella et al., 2019). Improving cropping options on saline soils include soil management and breeding for salt tolerant varieties (Flowers, 2004). There are multiple approaches of breeding for saline and sodic soils, including screening for existing genetic and physiological variation in under-developed crops, such as safflower.

Safflower (*Carthamus tinctorius* L.), a member of the Asteraceae family, is one of the oldest cultivated oilseed crop, grown in semi-arid and arid regions due to its stress tolerant nature (Emongor, 2010; Hussain et al., 2016; Singh and Nimbkar, 2016). An underutilized and underappreciated crop (Dajue and Mündel, 1996), safflower is currently only grown in 25 countries (FAO, 2018). A versatile crop, safflower not only has commercial and industrial uses, but also crucial agronomic benefits. Safflower forms deep root systems allowing penetration of compacted or sodic soils, improving soil structure, as well as accessing deep water and nutrient reserves, improving the growth of subsequent crops on otherwise marginal soils (Nuttall et al., 2008). Historically, safflower has been used as a vegetable, the source of the orange-red dye (carthamin), in traditional medicine, stock feed, and oil production (Dajue and Mündel, 1996; Singh and Nimbkar, 2016).

With the recent resurgence in renewable plant-based oils, interest in safflower has been renewed due to high oil yields (32–40%) and genotypic variation in fatty acid composition, in particular linoleic, stearic and monounsaturated oleic acids (Fernández-Martinez et al., 1993; Gecgel et al., 2006; La Bella et al., 2019). The oil is used for a range of applications including biofuel (Meka et al., 2007; de Oliveira et al., 2018), lubricants (Khemchandani et al., 2014), cosmetics (Wouters et al., 2010; Zemour et al., 2019), pharmaceuticals (Emongor, 2010; Asgarpanah and Kazemivash, 2013), food/cooking (Carvalho et al., 2006), and textiles (Wouters et al., 2010). Recent breeding efforts have focused on maximizing oil yields and increasing the oleic acid content, targeting expanding industrial markets (Anjani and Yadav, 2017; Wood et al., 2018; La Bella et al., 2019).

Safflower is a moderately salt (Francois et al., 1964; Maas, 1993; Kotuby-Amancher et al., 2000; Golkar, 2011) and drought tolerant (Istanbulluoglu, 2009; Hussain et al., 2016; La Bella et al., 2019) oilseed crop suitable for growing in a range of environments. Previous research has focussed on the effects of saline irrigation water, under field conditions, on safflower growth, morphology, and yield (Francois et al., 1964; Janardhan et al., 1986; Yeilaghi et al., 2015; Hussain and Al-Dakheel, 2018), as well as oil characteristics (Yuldasheva et al., 2011; Yeilaghi et al., 2012). Studies on glasshouse grown safflower have sought to understand the effects of Na^+^ on vital traits including salt tolerance inheritance (Golkar, 2011), stress signaling pathways (Severino et al., 2014; Shaki et al., 2019), and oil composition (Yuldasheva et al., 2011; Harrathi et al., 2013). Further studies have focused on the effects of salinity on seed germination and seedling vigor due to the particular sensitivity of safflower to Na^+^ at these earlier stages (Kaya et al., 2003, 2019; Ghazizade et al., 2012). While the above literature identified considerable variation in the salt tolerance of various safflower cultivars, no protocol has been developed to phenotype for salt response in an effective, reliable, and rapid manner.

High-throughput phenotyping is key in complementing recent advances in genomic breeding, especially through the use of rapid and high-throughput screening methods to screen for diversity among genotypes. The uptake of low-cost digital sensors and analysis algorithms has driven significant advances in plant phenotyping technology. High-throughput digital imaging has been used in a wide array of industries including forestry and agriculture, via a range of platforms, from satellites to unmanned aerial or ground-based vehicles, to hand-held sensors (Homolova et al., 2013; Fahlgren et al., 2015). Sensors and cameras measure spectral reflectance, the interaction of light and energy with plant components, at precise spectral regions including visible, often using red-green-blue (RGB; 400–700 nm), near infra-red (700–1,000 nm), and shortwave infrared (1,000–2,500 nm) (Li et al., 2014). Various imaging techniques for plant phenotyping have been developed to utilize spectral information including RGB, multispectral, hyperspectral, thermal, and fluorescence (Li et al., 2014). Despite the wide range of advanced digital phenotyping techniques, RGB imaging is often considered the most widely accessible and cost-effective method, due the comparatively lower cost of set-up, ease of maintenance, and variety of data output utilizations.

In recent years, advances in high-throughput digital imaging platforms in controlled environments have seen the rise of non-destructive data capture of plant traits, reducing the need for destructive measurements, and increasing the number of genotypes being screened. Controlled environment high-throughput phenotyping, using either plant-to-sensor or sensor-to-plant platforms, have been used to dissect plant traits including germination and early vigor (Nguyen et al., 2018), growth dynamics, biomass production or morphology (Golzarian et al., 2011; Neilson et al., 2015; Nguyen et al., 2019), and stress indicators (Sirault et al., 2009; Hairmansis et al., 2014; Neilson et al., 2015; Banerjee et al., 2020). Digital phenotyping has been used to dissect salt tolerance traits in a range of crops, including cereals (Krishnamurthy et al., 2007; Hairmansis et al., 2014; Takahashi et al., 2015; Tilbrook et al., 2017), pulses (Atieno et al., 2017), and grapevine (Henderson et al., 2018). These studies have shown that non-destructive digital estimations of plant growth, over multiple time points, consistently form high correlations with shoot fresh and dry weights (Golzarian et al., 2011; Hairmansis et al., 2014; Das Choudhury et al., 2018; Nguyen et al., 2018).

Here, we describe the development and application of a protocol for precise, high-throughput RGB digital phenotyping of salt tolerance in safflower at early vegetative growth stages, obviating the need to grow for the full lifecycle. We used the optimized protocol to screen 200 genotypically diverse safflower genotypes and to investigate mechanisms for salt tolerance in safflower. We highlighted the potential transferability of results obtained from glasshouse-based screening to field conditions. Our results show the protocol is an effective high-throughput approach for phenotyping diverse safflower genotypes for salt tolerance under controlled conditions, which, when coupled with high-throughput genomics, could be used to improve breeding of safflower varieties suited to saline soils.

## Materials and Methods

### Plant Materials, Growth Conditions, and Experimental Design

Plant Phenomics Victoria, Horsham is a state-of-the-art automated, high-throughput facility operated by Agriculture Victoria, Department of Jobs, Precincts and Regions. Detailed descriptions of the facility can be found in Banerjee et al. (2020). In brief, the Scanalyzer 3D plant-to-sensor platform (Lemnatec GmBH, Aachen, Germany), consists of a conveyor system with 600 carriers, automated weighing and watering stations, also used for salt application, and a digital imaging cabinet containing high-resolution RGB cameras.

The first experiment, using four released safflower (*C. tinctorius* L.) genotypes with differing oil composition and phenology (GRDC, 2017), Gila, Sironaria, S317, and Montola2003, was conducted with the aim to test and select salt treatments, using 0, 125, 250, and 350 mM sodium chloride (NaCl). Based on these results, the second experiment consisted of two salt treatments, 0 and 250 mM NaCl, to phenotype 200 diverse safflower genotypes (Supplementary Table 1), chosen to represent maximum genetic diversity in the Agriculture Victoria safflower collection.

For both experiments, Euro-TL white pots (200 mm diameter × 190 mm depth; Garden City Plastics, VIC, Australia) were filled by weight with 3.25 L of standard potting mix (Biogro, SA, Australia). Added to 1,000 L of standard potting mix were 3 kg Floranid N 32 IBDU (Compo GmbH & Co. KG, Münster, Germany), 5 kg Standard Brown Nutricote (Yates Australia, NSW, Australia), 3 kg Blue Colonizer Plus Macracote (Langley Fertilizer, WA, Australia), 1 kg MicroPlus Trace Element Fertilizer (Langley Fertilizer, WA, Australia), 225 g LiberFer SP Fe-chelate (BASF Corporation, NJ, United States), and 2 kg Debco SaturAid (Evergreen Garden Care Australia Pty Ltd, NSW, Australia) to ensure optimal plant nutrition. Pots were watered to pot capacity prior to sowing and placed on saucers throughout the experiment to prevent water/saline solution loss. Three seeds were sown per pot, then thinned to one seedling per pot 7 days after sowing (DAS) to ensure seedlings of uniform vigor across experiment. Plants were loaded onto the conveyer system at 15 DAS. The experiments were loaded in a complete randomized block design with up to six replicates per genotype per treatment. Growth conditions were controlled at 24/15°C day/night, with natural light conditions.

### Digital Imaging

**FIGURE 1:**
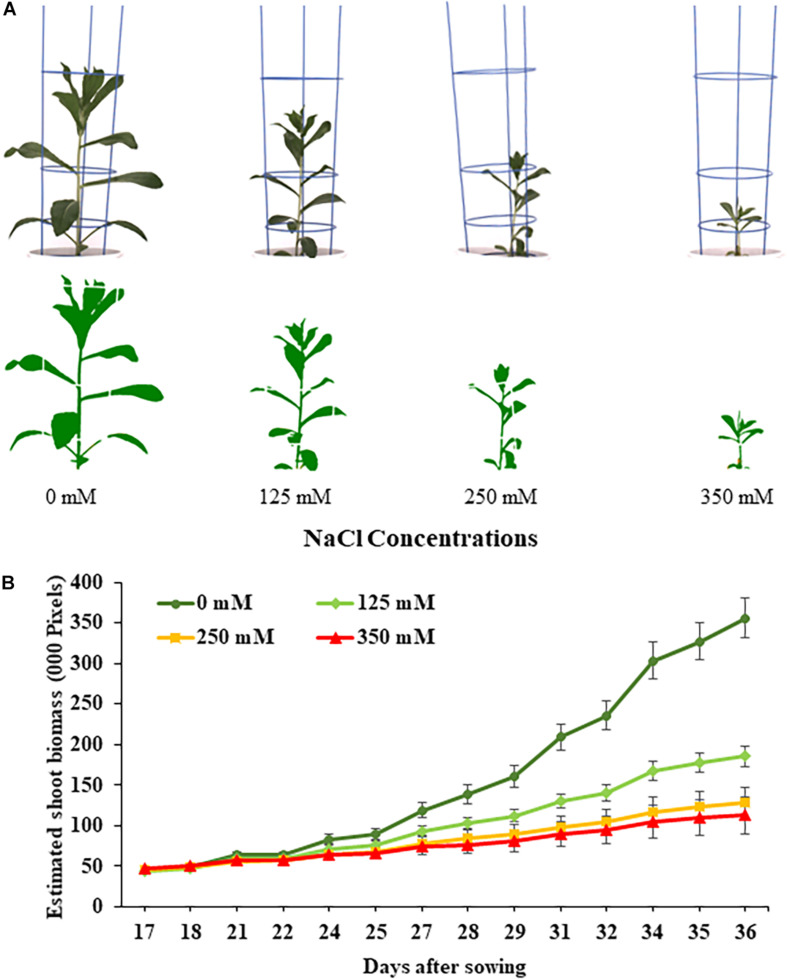
Performance of safflower genotypes under four salt (NaCl) concentrations from first experiment. **(A)** Raw and processed images of representative safflower plants at 36 days after sowing from the four salt treatments. **(B)** Growth curves showing the average performance, as estimated shoot biomass, of safflower genotypes across the growing period under four salt treatments. Data shown as mean with standard deviation. *n* = 96.

Digital RGB images were captured daily from 15 DAS until harvest at 36 DAS. Images were captured using two 28.8 Megapixel RGB cameras (top and side mounted), model Prosilica GT 6600C (Allied Vision Technologies, Stadtroda, Germany). Using the camera mounted directly above the plants, one digital RGB top view image was acquired. The three side view images were captured after consecutive rotations of the “turner” at 0°, 120°, and 240°. Captured images were automatically stored and analyzed in LemnaBase and LemnaGrid software (Lemnatec GmBH, Aachen, Germany). Details of image analysis pipelines used are described in Banerjee et al. (2020). In short, the region of interest consisting of all plant parts, was separated from the background of the raw images, then in subsequent steps image noise was removed and digital plant objects determined (Figure 1A). The pixel area from the four processed images per plant were then added together to calculate the estimated shoot biomass (ESB), digital plant volume, convex hull area, and plant height (PH).

### Salt Treatments

From 17 DAS, respective salt solutions were applied over several days to prevent salt shock, with 150 mL doses applied daily for 2 days for 0 and 125 mM, and for 3 days for 250 and 350 mM. To ensure calcium activity remained the same between control (0 mM) and salt treatments, 33 mM of CaCl_2_ was added to the 1M NaCl stock solution. This stock solution was diluted to achieve the correct application concentration for each salt treatment, based on the gravimetric soil water content (Supplementary Table 2). Saline solutions were administered through the saucer to prevent salt shock. Throughout the experiments, automated watering occurred to maintain pots at 5,300 *g*; the weight of pot, saucer, carrier, plant, and soil kept at 80% field capacity.

### Manual Destructive Harvesting

At 36 DAS all plants were destructively harvested. To determine fresh shoot biomass, plants were harvested at soil level and weighed. The third and fourth leaves (second leaf pair) and first and second youngest expanded leaves (youngest leaf pair) were removed, weighed separately and put into separate 15 mL tubes. The fresh biomass and two leaf pairs were dried at 70°C for 3 days, then weighed to obtain dry biomass. Leaf pair samples were used for ion analysis.

### Ion Analysis of Leaf Tissue and Soil

Leaves were digested in 1% (v/v) nitric acid at 100°C for 4 h in a water bath (TWB-48D; Thermoline Scientific Equipment Pty. Ltd., NSW, Australia). The Na^+^ and potassium (K^+^) concentrations of the digested leaves were determined using a flame photometer (Sherwood 420, Sherwood Scientific, Cambridge, United Kingdom).

Soil from four pots per treatment from the first experiment were sampled at the end of the experiment to determine the Na^+^ concentration of potting mix at different depths. The pots were divided into three depths, 0–5, 6–10, and 11–15 cm, dried for 3 days at 70°C, then subsampled. Na^+^ and K^+^ concentrations were measured using a 1:5 (soil:water) extract, after samples had been shaken on an orbital shaker for 2 h and settled for 1 h. Concentrations were determined using flame photometry.

### Field Trial Under Sodic Soil Conditions

Based on the results from the second glasshouse experiment, eight genotypes (two salt sensitive, two moderately salt tolerant, and four salt tolerant) were chosen to grow in a field trial on sodic soil at Lockhart, NSW, Australia (35°14′27.46″ S, 146°48′11.07″ E) from May 2019 to January 2020. The field site was a slightly sodic red-brown loam soil, with 6% exchangeable Na^+^ percentage to 80 cm depth. The genotypes were sown using a disk seeder at a rate of 35 seeds/m^2^, in six rows per 2 m × 12 m plot, in three blocks using a randomized complete block design. During sowing MAP (60 kg/ha) and Granam (50 kg/ha) fertilizers were applied. Normal agronomic practices were followed during the season. The trial received 245 mm of rainfall during the growth season, well below the average rainfall for this period of 413 mm.

At 48 DAS, plant count (per meter) and vigor (1–9 scale) observations were recorded. Once plots were machine harvested, seed yield (tons/hectare) was obtained and yield (g/plant) was calculated using final plant counts. Salinity rankings were determined by comparing the eight genotypes to each other, with classification at 48 DAS based on a combination of plant count and vigor.

### Statistical Analysis

The salt tolerance of safflower genotypes was calculated using a salt tolerance index (STI; Negrão et al., 2016), based on the ESB of a genotype under control (b_c_) and salt treatments (b_s_), using the formula: *STI* = b_s_/b_c_. Plant were grouped as strongly salt sensitive, salt sensitive, salt tolerant, or strongly salt tolerant based on STI, in a similar method to Ahmad et al. (2013). Salt sensitive genotypes were broken into two further classes, as strongly salt sensitive genotypes had a 50–95% reduction in biomass under salt treatment, while salt sensitive genotypes had 10–49% reductions in biomass. Salt tolerant genotypes were also further classified into two classes, with salt tolerant genotypes maintaining growth under salt treatment, with a 10–20% leeway either side, while strongly salt tolerant genotypes had over 10% increases in biomass production. Pearson’s correlation coefficient and *t*-tests were performed to assess the relationship between digital biomass-related indices and ion-content indices from the second experiment. Similar to STI, other indices were calculated for digital traits; digital volume index (DVI), convex hull index (CHAI), plant height index (PHI), and salt tolerance index derived from manual dry biomass (STI-DB). Ion content changes and ratios were also calculated, i.e., the change in Na^+^ content in the third and fourth leaves (Na^+^ third and fourth), the change in Na^+^ content in the first and second youngest leaves (Na^+^ first and second), the K^+^ to Na^+^ ratio at 250 mM treatment in the third and fourth leaves (K^+^/Na^+^ third and fourth), and the K^+^ to Na^+^ ratio at 250 mM treatment in the first and second youngest leaves (K^+^/Na^+^ first and second).

## Results

### Analysis of Safflower Growth Using Digital Imaging

**FIGURE 2:**
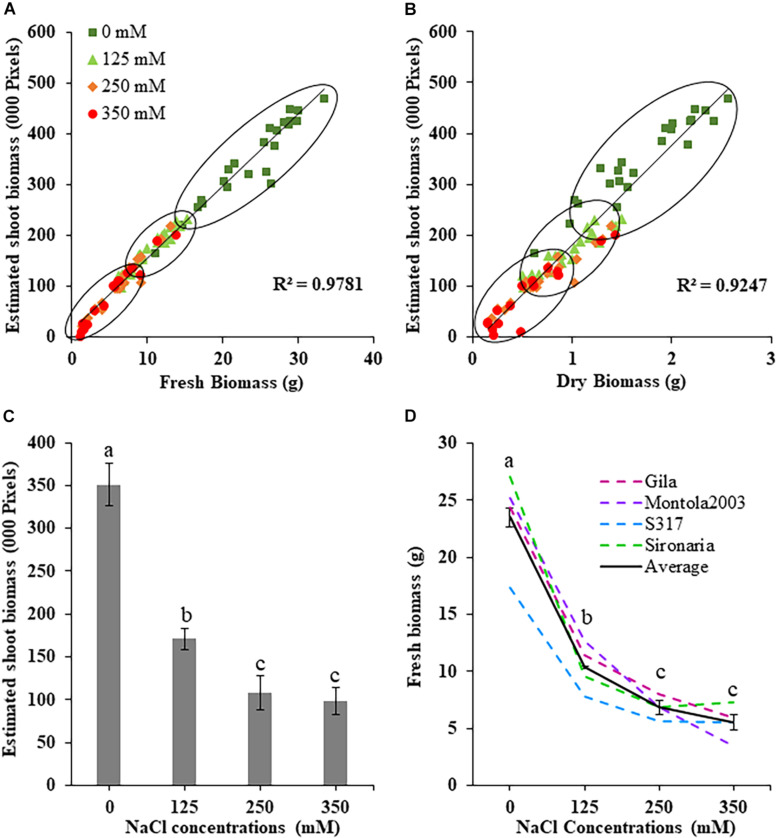
Measured and estimated shoot biomass in safflower genotypes at four salt (NaCl) concentrations from first experiment. **(A)** Correlation between estimated shoot biomass and fresh biomass and **(B)** dry biomass harvested at 36 days after sowing. **(C)** Estimated shoot biomass accumulation for all genotypes at the four salt concentrations. **(D)** Fresh biomass at the four salt concentrations for the four safflower genotypes and average of all genotypes. Data represents mean and standard deviation. *n* = 96. Different letter (a,b,c) indicate a significant difference (*P* < 0.05) for fresh biomass at different salt level.

The effects salt treatments had on safflower plant growth is illustrated in Figure 1, with reduced biomass production, especially at 250 and 350 mM. A week after salt treatment was applied, ESB growth curves for the four treatments began to noticeably separate, with significantly large differences noticeable from 29 DAS onward (Figure 1B). Very strong correlations were observed between ESB and shoot fresh (*R*^2^ = 0.978) and dry (*R*^2^ = 0.925) weights, with separation between control, 125 and 250/350 mM plants (Figures 2A,B). When comparing biomass from 200 diverse safflower genotypes grown in the second experiment, strong correlations were observed between ESB and shoot fresh (*R*^2^ = 0.828) or dry (*R*^2^ = 0.725) weights (Supplementary Figure 1). ESB showed significant differences in biomass between treatments (Figure 2C), in a similar trend to fresh biomass of all four genotypes (Figure 2D, solid black line). Variation in biomass production between genotypes (Figure 2D), likely explained some overlap between groups in Figures 2A,B.

### Defining Salinity Stress Levels in Safflower

**FIGURE 3:**
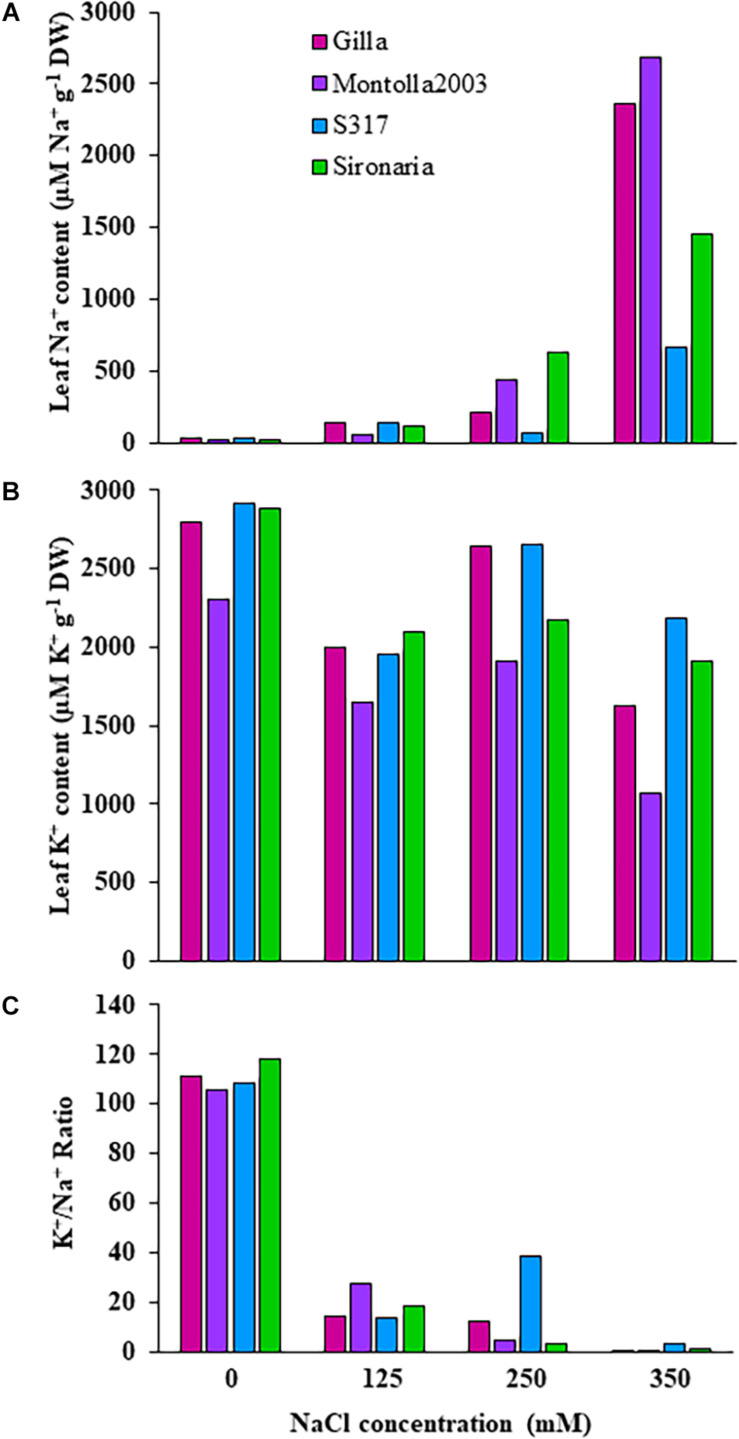
Ion contents in third and fourth leaves of four safflower genotypes under four salt (NaCl) treatments from first experiment. **(A)** Sodium content, **(B)** potassium content, and **(C)** K^+^/Na^+^ ratio in third and fourth leaves of four safflower genotypes at 36 days after sowing.

The first experiment used four safflower genotypes grown under four salt treatments (0, 125, 250, and 350 mM) to define the salt stress levels for further phenotyping in safflower. Saline solutions were applied to the saucer to prevent salt shock, and ensure the middle level of pots, where roots were most concentrated, reached the defined salt concentrations (Supplementary Figure 2). Control plants (0 mM) showed considerable growth at 36 DAS, with expected very low Na^+^ and high K^+^ concentrations (Figures 1, 3). While plants at 125 mM had a mild drop in biomass, there was still overlap in both fresh and dry biomass with control plants, due to genotypic variation (Figures 2A,B). Plants at 350 mM suffered a significantly severe drop in biomass compared to control plants for all genotypes and showed signs of necrosis (Figures 1A, 2C). This correlated to the highest uptakes of Na^+^ and large drops in K^+^ severely effecting the K^+^/Na^+^ ratio (Figure 3). Significant differences between genotypes were observed, with genotype Montola2003 the most affected, taking up the highest or second highest concentrations of Na^+^, and lowest levels of K^+^ at both 250 and 350 mM treatments (Figure 3). While plants grown at 250 mM, also showed significant drops in biomass, and had high Na^+^ concentrations, differences could be seen between genotypes without plants health being severely affected (Figures 1–3). These results demonstrated that 125, 250, and 350 mM showed mild, moderate, and severe effects on safflower growth. Therefore, 250 mM NaCl was chosen for further experiments, to ensure differences could be identified between diverse genotypes.

### Comparative Performance of Diverse Safflower Genotypes

**FIGURE 4:**
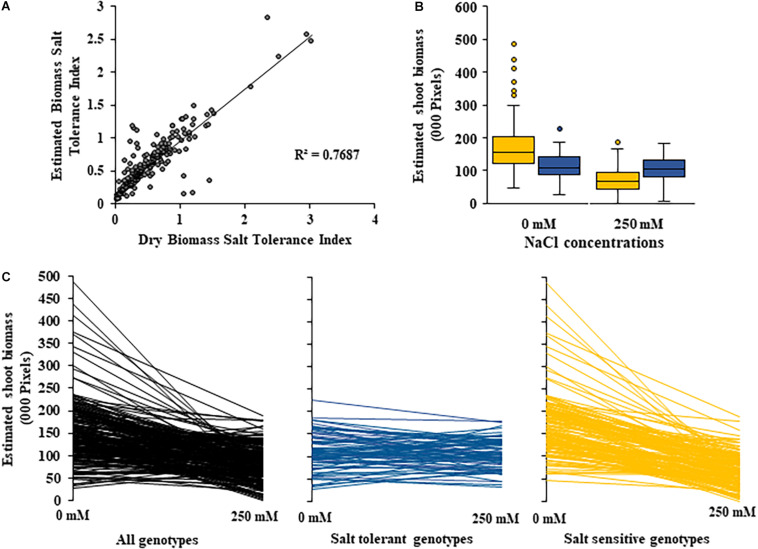
Changes in shoot biomass between control and salt (NaCl) treatments for 200 diverse safflower genotypes. **(A)** Correlation between estimated biomass and dry biomass salt tolerance index. **(B)** Boxplots showing spread of estimated shoot biomass data for safflower genotypes classified as salt tolerant (blue) or salt sensitive (yellow) based on dry biomass salt tolerance index. Boxplot plots represent minimum, maximum and mean values as well as interquartile range and outliers. **(C)** Estimated shoot biomass under control (0 mM) and salt (250 mM) treatments for 200 diverse safflower genotypes. Black – all genotypes; blue – salt tolerant genotypes *n* = 65; and yellow – salt sensitive genotypes *n* = 135.

**FIGURE 5:**
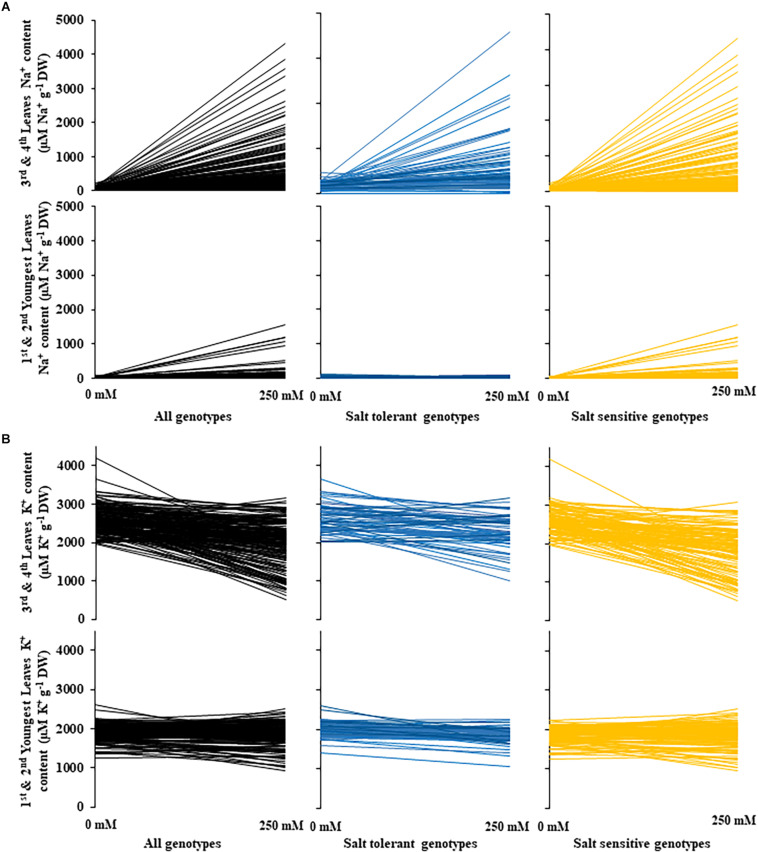
Changes in sodium and potassium leaf content between control and salt (NaCl) treatments for 200 diverse safflower genotypes. **(A)** Sodium content of safflower genotypes under control (0 mM) or salt (250 mM) treatments; sodium content of the third and fourth leaves in the upper panel and sodium content of the first and second youngest expanded leaves in the lower panel. **(B)** Potassium content of safflower genotypes under control (0 mM) or salt (250 mM) treatments; potassium content of the third and fourth leaves in the upper panel and potassium content of the first and second youngest expanded leaves in the lower panel. Black – all genotypes; blue – salt tolerant genotypes; and yellow – salt sensitive genotypes.

**FIGURE 6:**
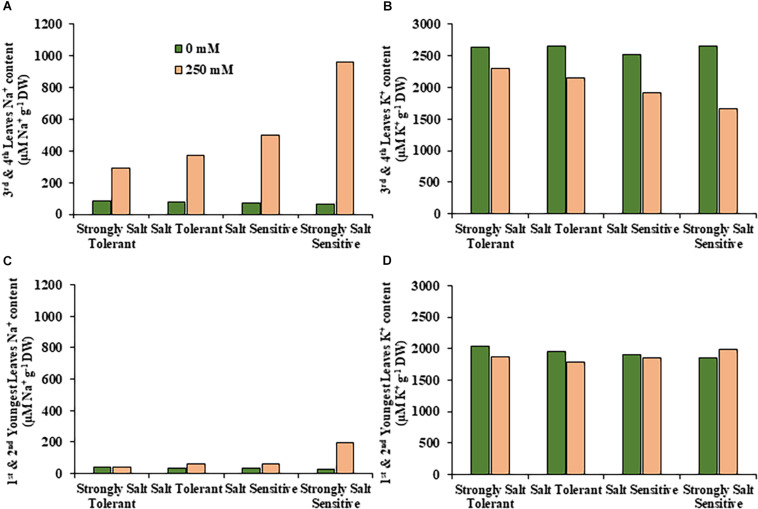
Average sodium and potassium content in leaves for salt tolerant and salt sensitive safflower genotypes under control and salt (NaCl) treatments. Sodium content of significantly salt tolerant, salt tolerant, salt sensitive and significantly salt sensitive genotypes for **(A)** third and fourth leaves and **(C)** first and second youngest expanded leaves under control (0 mM; green) and salt (250 mM, pink) treatments. Potassium content of strongly salt tolerant, salt tolerant, salt sensitive, and strongly salt sensitive genotypes for **(B)** third and fourth leaves and **(D)** first and second youngest expanded leaves under control (0 mM; green) and salt (250 mM, pink) treatments.

**FIGURE 7:**
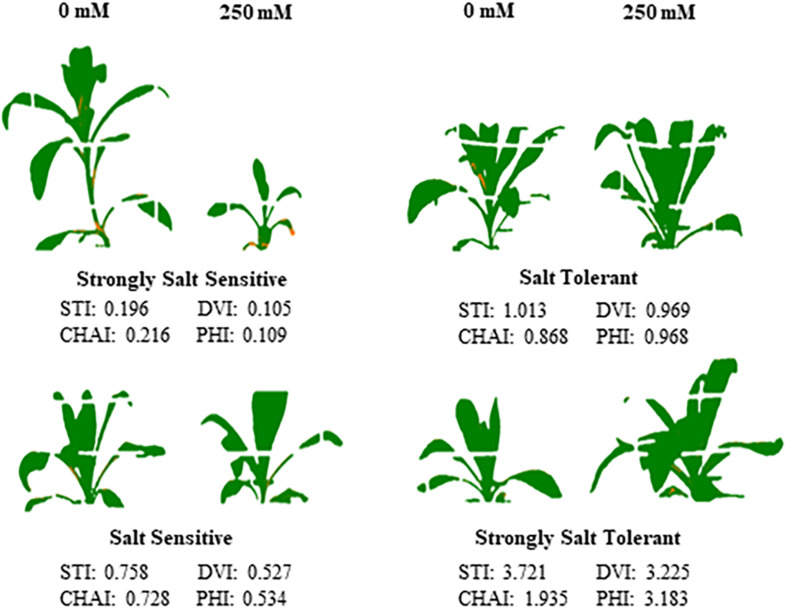
Performance of the four classes of safflower genotypes under control and salt (NaCl) treatments. Processed images and dry biomass salt tolerance index (STI) of genotypes under control (0 mM) and salt (250 mM) treatments, representing the four classes of genotypes; strongly salt sensitive (STI < 0.5), salt sensitive (STI 0.5–0.8), salt tolerant (0.8–1.1), and strongly salt tolerant (>1.1).

A moderate correlation (*R*^2^ = 0.769; *r* = 0.604) was observed between the STI calculated using ESB and dry biomass (Figure 4A and Supplementary Figure 3). High correlations were observed between STI-ESB and DVI (*r* = 0.957), CHAI (*r* = 0.939), and PHI (0.963). STI-DB had significant, moderate correlations with the same traits (Supplementary Figure 3). Based on the STI-ESB, all 200 genotypes were classified as either salt tolerant or salt sensitive. 65 genotypes essentially maintained biomass or increased biomass production under salt treatment, while 135 genotypes suffered biomass loss under saline conditions (Figure 4B). The change in biomass production between the two treatments is shown in Figure 4C. Interestingly, genotypes which performed best under control conditions were the most salt sensitive. The classification of genotypes based on STI-ESB corresponds with changes in ion content. Overall, salt tolerant genotypes maintained low or had only slight increases in Na^+^ content in both the second and youngest leaf pairs, coupled with the maintenance or small K^+^ reductions in both leaf sets (Figures 5, 6). However, salt sensitive genotypes showed moderate to very large increases in Na^+^ content in both the second and youngest leaf pairs, as well as large decreases in K^+^ in the second leaf pair (Figures 5, 6). To further elucidate the biomass and ion content trends associated with safflower salt sensitivity or tolerance, genotypes were further divided into four groups based on STI; strongly salt sensitive, salt sensitive, salt tolerant, strongly salt tolerant (examples in Figure 7), which corresponded to leaf Na^+^ and K^+^ levels. Strongly salt sensitive genotypes had large reductions in biomass, corresponding to extremely high Na^+^ in the second leaf pair, relatively high Na^+^ content in the youngest leaves, and large decreases in K^+^ content in the second leaf pair (Figure 6). Salt sensitive genotypes showed similar trends, with high Na^+^ content and lower K^+^ content in the second leaf pair but only a small rise in Na^+^ content in the youngest leaf pair (Figure 6). Salt tolerant genotypes had a moderately high Na^+^ content and a moderate decrease in K^+^ in the second leaf pair, with only a small rise in Na^+^ content in the youngest leaf pair (Figure 6). Strongly salt tolerant genotypes had a moderate rise in Na^+^ levels and a small decrease in K^+^ in the second leaf pair, with almost no differences to control plants in the youngest leaf pair (Figure 6). Interestingly, the change in Na^+^ content in the first and second youngest leaves shown moderately low correlations with biomass based trait indices STI-ESB (*r* = 0.238), CHAI (*r* = 0.334), and STI-DB (*r* = 0.264), while change in Na^+^ content in the third and fourth leaves, showed no correlation to any traits (Supplementary Figure 3). The performance of example genotypes for each of the four STI-based classifications are given in Figure 7.

### Field Evaluation of Eight Genotypes

To understand if the salt tolerance or sensitivity classifications determined using glasshouse-based phenotyping showed any correlation to performance in the field, eight genotypes, classified as salt sensitive, moderately salt tolerant or salt tolerant, were grown under sodic field conditions. During the field trial, at 48 DAS (two leaf pairs fully emerged), genotypes were ranked into the three salt tolerance categories based on a combination of plant establishment observations, counts, and vigor scores. Observations taken in the field at 48 DAS, were equivalent to those taken when plants were approximately 20 DAS under glasshouse environments, when plants also had two leaf pairs fully emerged. From the establishment observations, five genotypes were found to maintain the same ranking at 48 DAS between field and glasshouse experiments, one found to perform better and two performed worse (Table 1). Based on seed yield results, five genotypes maintained ranking, one performed better and two performed worse (Table 1). While some genotypes (i.e., S317, Sironaria, AVS-SAFF-228, and AVS-SAFF-56) maintained salinity ranking across their full lifecycles, other genotypes (SIGMA46 and AVS-SAFF-247) were found to differ majorly in salt tolerance between young plants and yield in the field experiment.

**TABLE 1:** Performance of safflower genotypes grown under sodic field conditions compared to performance in glasshouse.

**Variety**	**Emergence (plants/m^2^)**	**Vigor**	**Yield (g/plant)**	**Ranking at 48 DAS**	**Ranking at harvest**	**Glasshouse ranking**
SIGMA46	18.8	5.3	2.081	Salt sensitive	Salt tolerant	Salt sensitive
AVS-SAFF-228	38.8	6.7	0.690	Moderately salt tolerant	Moderately salt tolerant	Salt sensitive
Sironaria	37.0	6.3	0.545	Moderately salt tolerant	Moderately salt tolerant	Moderately salt tolerant
S317	38.4	6	0.762	Moderately salt tolerant	Moderately salt tolerant	Moderately salt tolerant
AVS-SAFF-247	19.2	4	1.702	Salt sensitive	Salt tolerant	Salt tolerant
Hamaya65	24.6	7.3	0.916	Moderately salt tolerant	Salt tolerant	Salt tolerant
AVS-SAFF-56	34.1	7	0.948	Salt tolerant	Salt tolerant	Salt tolerant
AVS-SAFF-18	40.9	7.7	0.645	Salt tolerant	Moderately salt tolerant	Salt tolerant

*DAS; Days after sowing.*

## Discussion

Increasing salt tolerance will play an important role in improving the growth, development, and yields of crops grown on the ever increasing areas of saline soils and reopening cropping opportunities on salinized lands (Ivushkin et al., 2019). Modern breeding efforts, which have identified different salt tolerance mechanisms (Munns and Tester, 2008; Roy et al., 2014), rely on innovative and high-throughput genotyping and phenotyping platforms to identify tolerant germplasm. Historically, conventional phenotyping for salt tolerance often involved hydroponic set-ups, manual Na^+^ measurements, and destructive harvesting (Munns and James, 2003; Genc et al., 2007; Javed et al., 2014).

Therefore, the integration of technology which can improve cost and time efficiencies, such as image-based, non-destructive phenotypic sensors, is key to improving crop breeding programs (Fahlgren et al., 2015). Image-based phenotyping has been used in previous salinity studies to dissect tolerance mechanisms and screen germplasm in pulses (Atieno et al., 2017) and a range of cereals (Krishnamurthy et al., 2007; Hairmansis et al., 2014; Takahashi et al., 2015; Tilbrook et al., 2017). In this study, we report a novel protocol for high-throughput, image-based salt tolerance screening of safflower, during early vegetative stages, allowing for the rapid phenotyping of large germplasm populations. Repetitive, non-destructive phenotypic measurements have previously been demonstrated to enable analysis of plant traits across growth stages, through the mapping of growth curves (Meng et al., 2017; Das Choudhury et al., 2018; Nguyen et al., 2018, 2019). Our results show that overall, high-throughput image-based phenotyping techniques can be used to screen large germplasm populations, and identify candidate genotypes for further field evaluations. This protocol fits with previous research which has also demonstrated that early vegetative screens can provide insight into the performance and yield of germplasm at later stages (Krishnamurthy et al., 2007; Meng et al., 2017) or under stresses (Nguyen et al., 2019; Banerjee et al., 2020).

From RGB images captured during high-throughput phenotyping in this study, plant biomarker ESB was calculated over the 20 day imaging period for each genotype. High linear correlations were found between ESB, and plant fresh and dry biomass, similar to those seen in other crops (Golzarian et al., 2011; Hairmansis et al., 2014; Nguyen et al., 2018; Banerjee et al., 2020). This suggests that ESB can be effectively used to estimate fresh and dry biomass, obviating the need for destructive harvesting. Biomass at vegetative stages, under abiotic stresses, have been shown to highly correlate to biomass production at maturity, illustrating that performance in vegetative screens is a good indicator of performance at yield (Nguyen et al., 2019; Banerjee et al., 2020). Biomass-based traits show high narrow-sense heritability, although they are controlled by additive gene effects, and strong links to yield performance, making them strong selection parameters in early vegetative screens (Moragues et al., 2006; Golkar, 2011; Yeilaghi et al., 2015). Therefore, since biomass-based parameters, ESB, fresh or dry weights, and STIs, are determining factors of salt tolerance, the selection of genotypes, based on these traits could be highly effective under saline conditions (Golkar, 2011; Yeilaghi et al., 2015).

Previous work on safflower has shown little change in growth parameters below 100 mM Na^+^, with large reductions in biomass seen above 150 mM (Harrathi et al., 2013; Singh et al., 2013; Gengmao et al., 2015), although young seedlings are more sensitive at lower concentrations (Kaya et al., 2003, 2019; Ghazizade et al., 2012). In the first experiment plants grown under 125 mM NaCl were shown to have a 50% decrease in plant biomass (PH and digital volume), across all genotypes, as well as increased shoot Na^+^ and decreased shoot K^+^ levels. Meanwhile, 250 and 350 mM Na^+^ had moderate to severe effects on plant growth (nearly 75% biomass drop), with higher increases in shoot Na^+^, decreases in K^+^, and extremely low K^+^/Na^+^ ratios. These findings align with the characteristics of safflower as a moderately salt tolerant crop, with 50% biomass loss, at 125 mM NaCl (12.5 dS/m), and severely impacted growth at 250 mM (25 dS/m) and 350 mM (35 dS/m; Janardhan et al., 1986; Maas, 1993; Kotuby-Amancher et al., 2000). Interestingly, genotypic variation was mainly seen in the higher NaCl treatments supporting the idea that to identify stress tolerance variation in populations of diverse germplasm, moderate to severe stress conditions are ideal.

Plants respond and adapt to Na^+^ toxicity in a myriad of ways which can be categorized as either shoot ion independent or shoot ion dependent pathways. Shoot ion independent tolerance involves the rapid regulation of long-distance sensing and signaling of salt stress, triggering responses, including the reduction of growth, and production of protective osmolytes and secondary metabolites to regulate osmotic and leaf water potentials (Munns and Tester, 2008; Roy et al., 2014; Hussain et al., 2016). Shoot ion dependent tolerance mechanisms come into effect days after initial stress and revolve around the movement of Na^+^ across membranes. Ion exclusion pathways operate in the roots and vascular system, moving and removing Na^+^, either completely out of plants at the root surface or removing it from circulating to sensitive tissues (Munns and Tester, 2008; Roy et al., 2014). Tissue tolerance, the sequestrations of Na^+^ into the vacuole from the cytosol and synthesis of compatible solutes, allows plants to deal with Na^+^ which has reached leaf tissue (Munns and Tester, 2008; Roy et al., 2014). The above mechanisms are not mutually exclusive, but rather the dominance of each tolerance mechanisms switches under different circumstances (Roy et al., 2014; Hussain et al., 2016).

Safflower appears to have strong osmotic tolerance mechanisms when grown in environments with less than 100 mM Na^+^, being able to synthesis a range of compatible solutes and secondary metabolites for osmotic adjustments and preservation of leaf water potential (Singh et al., 2013; Javed et al., 2014; Gengmao et al., 2015; Hussain et al., 2016). At higher salinity levels, safflower seems to rely on ion exclusion and tissue tolerance mechanisms. Safflower roots have been shown to sequester high concentrations of Na^+^ and Cl^–^ in the roots, suggesting that safflower is able to partition toxic ions away from sensitive leaf tissue (Patil, 2012; Karimi et al., 2014). In this study, Na^+^ accumulation increased with the salt concentration, with genotypic differences more prominent at 250 and 350 mM, i.e., S317 accumulating lower Na^+^ and higher K^+^ than other varieties. This fits with previous research showing that while Na^+^ accumulation in safflower leaves increased with stress levels, salt tolerance was linked to genotypes which uptake less Na^+^ and more K^+^ compared to sensitive genotypes (Hosseini et al., 2010; Harrathi et al., 2013; Karimi et al., 2014; Yeilaghi et al., 2015).

To allow for further dissection of the likely salt tolerance mechanisms used by safflower, the diverse safflower population was divided into four classes of tolerance or sensitivity based on their STI-ESB. While no significant differences were seen between groups, due to the variation in responses to salt between genotypes, clear trends were observed as follows: salt tolerant and strongly salt tolerant genotypes tend to produce moderate biomass under control conditions and were able to maintain or produce higher biomass production under salt stress; salt sensitive and strongly salt sensitive genotypes suffered severe reductions in biomass under salt stress, although interestingly some of the more sensitive genotypes were the best performing of all genotypes under control conditions. This demonstrates why germplasm selected only in control condition screens, will often produce poor performance in more realistic stress environments (Rosielle and Hamblin, 1981).

Due to the complex nature of salt tolerance responses, ion accumulation has been reported as both connected (Yeilaghi et al., 2015) and detached from growth parameters (Genc et al., 2007; Tilbrook et al., 2017). Although individual genotypes had different ion accumulation profiles, clear trends for each of the four salt tolerance or sensitivity classes were identified, which matched their biomass production. Genotypes from all classes had very little differences in Na^+^ uptake under control conditions, although K^+^ accumulation differed, which may explain some of the differences in growth, as K^+^ is a macronutrient vital for plant growth. Tolerant genotypes, which produced more biomass under 250 mM NaCl conditions, typically showed low levels of Na^+^ accumulation and only a small decrease in K^+^ in the second leaf pair, as well as very little change in accumulation of either Na^+^ or K^+^ in the youngest leaf pair. Salt tolerant genotypes, which maintained biomass at 250 mM NaCl, show similar uptake patterns, although more Na^+^ was accumulated in both leaf pairs. These results, as well as the significant low correlations between biomass indices and Na^+^ in the youngest leaves, suggest salt tolerance in safflower is dependent on ion exclusion, especially in the youngest growing tissue, likely resulting in strong root tolerance and exclusion mechanisms. These genotypes also maintained a higher K^+^/Na^+^ratio in both leaf pairs, consistent with the behavior of other salt tolerance genotypes identified in previous studies (Hosseini et al., 2010; Karimi et al., 2014; Yeilaghi et al., 2015).

Salt sensitive genotypes, which showed moderate reductions in biomass at 250 mM NaCl, accumulated even more Na^+^ and less K^+^ in the second leaf pair, although they maintained similar ion content in the youngest leaf pair as salt tolerant plants. These genotypes likely had a reduced ability to exclude Na^+^ from leaf tissue. Strongly salt sensitive genotypes, which had the largest reductions in biomass at 250 mM NaCl, showed the highest Na^+^ accumulation in both leaf pairs, as well as the lowest K^+^ uptake in the second leaf pair. These genotypes likely have poor abilities to excluded Na^+^ throughout the plant, but especially in leaf tissue. Overall, results suggest that salt tolerance in safflower at high salt concentrations, is highly dependent on the exclusion of Na^+^ from all shoot tissue, likely through transport proteins including SOS1 and NHX1 (Munns and Tester, 2008; Roy et al., 2014; Shaki et al., 2019), allowing biomass production to continue at near normal rates. Tolerance in safflower is also likely achieved through strong osmotic tolerance mechanisms, including changing the composition profile of sugars (Javed et al., 2014; Gengmao et al., 2015), antioxidants (Hosseini et al., 2010; Yuldasheva et al., 2011), osmolytes (Karimi et al., 2014; Gengmao et al., 2015), and fatty acids (Yuldasheva et al., 2011; Yeilaghi et al., 2012; Harrathi et al., 2013; Javed et al., 2014).

Continuing from the glasshouse studies conducted in this study, eight genotypes representing three of the classes (salt sensitive, salt tolerant/moderately salt tolerant, and strongly salt tolerant/salt tolerant) were grown in a field trial with sodic soil conditions. Interestingly, five of the eight genotypes in field experiment performed in a similar or only slightly different manner when compared to their glasshouse performance. Three genotypes, Sironaria, S317 and AVS-SAFF-56, had consistent performance in both glasshouse and field conditions based on biomass, vigor, and yield scores. Previous studies have shown performance consistencies between glasshouse and field screens (Schilling et al., 2014; Pardo et al., 2015; Peirone et al., 2018). The salt tolerance of other genotypes, SIGMA46, AVS-SAFF-247, and AVS-SAFF-18, varied depending on the stage of development in the field, often showing reduced vigor, but comparatively higher yields. Differences in performance between glasshouse and field are likely due to environmental factors, such as the presence of salt at germination, and soil environment differences due to the sodic nature of the site, which have been known to affect safflower growth (Kaya et al., 2003; Ghazizade et al., 2012; Severino et al., 2014). Inconsistent performance between glasshouse and field studies has been documented, due to variations in competition, environmental factors, inconsistent stress application, and soil type (Araus and Cairns, 2014; Junker et al., 2014). Vegetative phenotypic screens, like those performed in this study, can therefore be useful indicators of the likely salt tolerance of genotypes and their potential performance under field conditions.

## Conclusion

In conclusion, the vegetative screening method presented here demonstrates the use of biomass-based salt tolerance indices in explaining the salt tolerance of safflower genotypes and predict their performance under field conditions. Our findings show that high-throughput digital RGB imaging can be used to effectively differentiate salt tolerant and salt sensitive safflower genotypes. Here, we also demonstrate that at high salt concentrations, safflower relies on Na^+^ exclusion and maintenance of K^+^/Na^+^ ratios to infer salt tolerance. Consistent performance of a few representative genotypes under both glasshouse and field conditions demonstrated that this protocol, and vegetative screens in general, can be useful in predicting potential performance under field conditions. Further research is needed to elucidate the potential for vegetative screening protocols to predict potential field performance. This protocol provides a robust assessment tool for safflower populations, enabling the rapid identification of candidate germplasm to enhance salt tolerance.

## Data Availability Statement

The original contributions presented in the study are included in the article/Supplementary Material, further inquiries can be directed to the corresponding author/s.

## Author Contributions

SK, MH, HD, and GS conceived the research plan. SK, ET-K, and SJ conceptualized and planned the experiments. ET-K and SJ conducted the experiments. ET-K performed data analysis and wrote the first draft of the manuscript. DH conducted the field trial under sodic conditions. ET-K, SK, SJ, MH, HD, DH, and GS contributed to manuscript editing, revision, reading and approval of the submitted version. All authors contributed to the article and approved the submitted version.

## Conflict of Interest

DH was employed by the company GO Resources Pty Ltd. The remaining authors declare that the research was conducted in the absence of any commercial or financial relationships that could be construed as a potential conflict of interest.
