# Supplementary material for: Digital Phenotyping to Delineate Salinity Response in Safflower Genotypes
Source: Front Plant Sci. 2021 Jun 16;12:662498. doi: 10.3389/fpls.2021.662498 (PMC8242588; doi:10.3389/fpls.2021.662498)
Supplement: Supplementary file 1 [file Presentation_1.PPTX]

## Slide 1
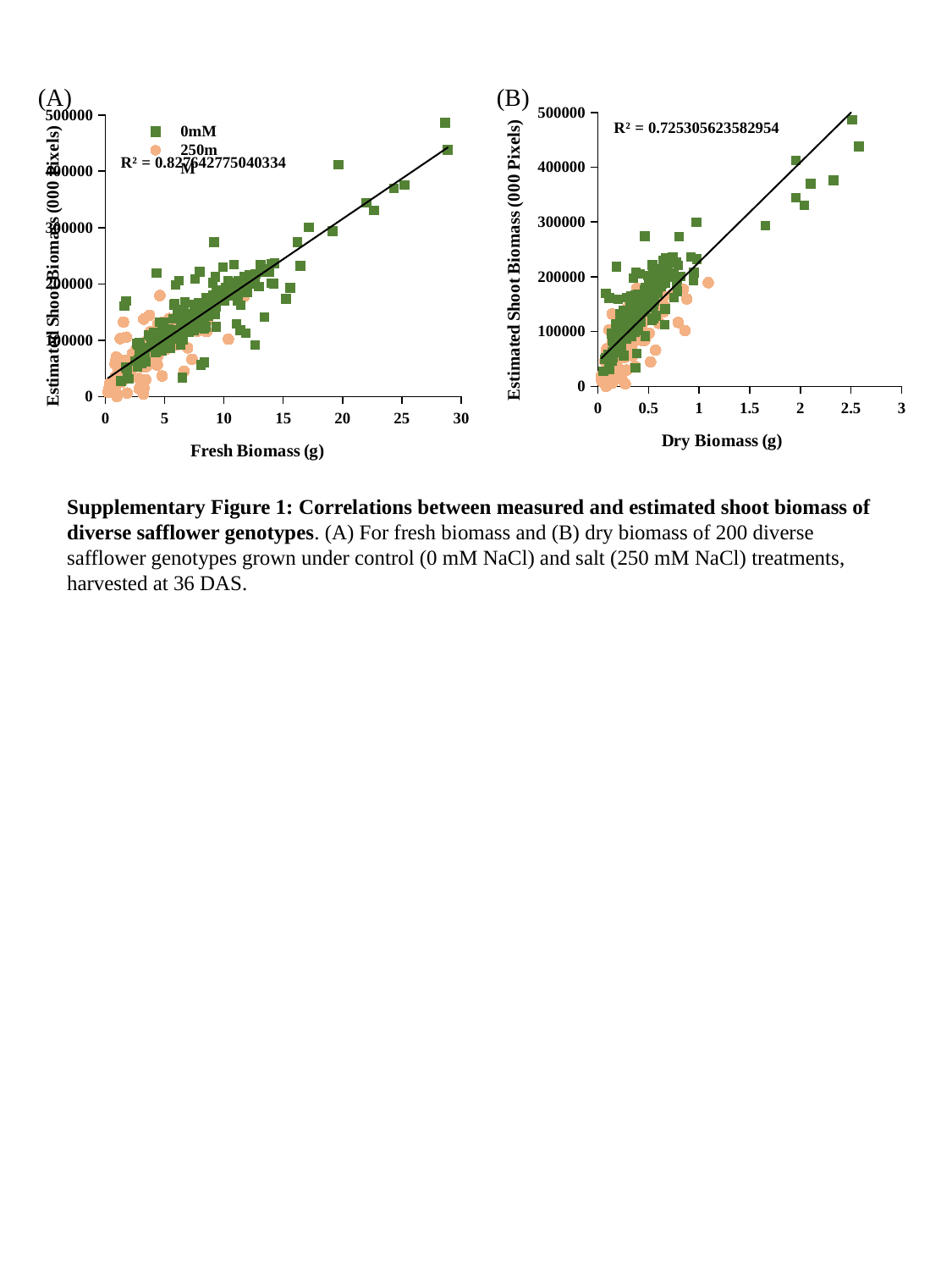

(B)
(A)
### Chart
| Category | | | |
|---|---|---|---|
### Chart
| Category | | | |
|---|---|---|---|Supplementary Figure 1: Correlations between measured and estimated shoot biomass of diverse safflower genotypes. (A) For fresh biomass and (B) dry biomass of 200 diverse safflower genotypes grown under control (0 mM NaCl) and salt (250 mM NaCl) treatments, harvested at 36 DAS.

## Slide 2
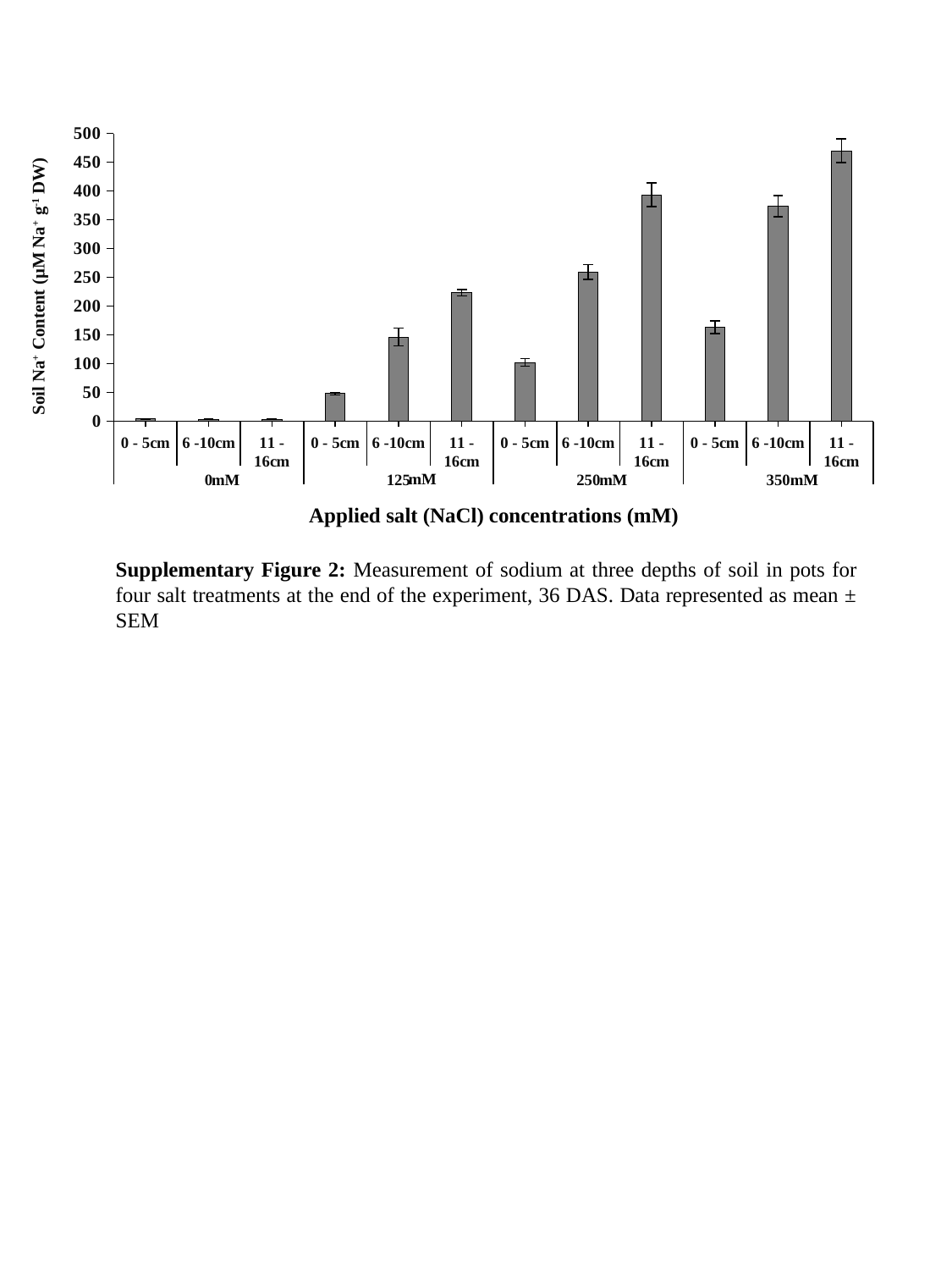

### Chart
| Category | |
|---|---|
| 0 - 5cm | 4.1421875 |
| 6 -10cm | 3.3359375 |
| 11 -16cm | 3.8140625 |
| 0 - 5cm | 48.041666666666664 |
| 6 -10cm | 146.53333333333333 |
| 11 -16cm | 223.36666666666667 |
| 0 - 5cm | 102.25 |
| 6 -10cm | 259.45 |
| 11 -16cm | 393.50000000000006 |
| 0 - 5cm | 163.4 |
| 6 -10cm | 373.79999999999995 |
| 11 -16cm | 470.2 |Soil Na+ Content (µM Na+ g-1 DW)
mM
mM
mM
mM
Applied salt (NaCl) concentrations (mM)
Supplementary Figure 2: Measurement of sodium at three depths of soil in pots for four salt treatments at the end of the experiment, 36 DAS. Data represented as mean ± SEM

## Slide 3
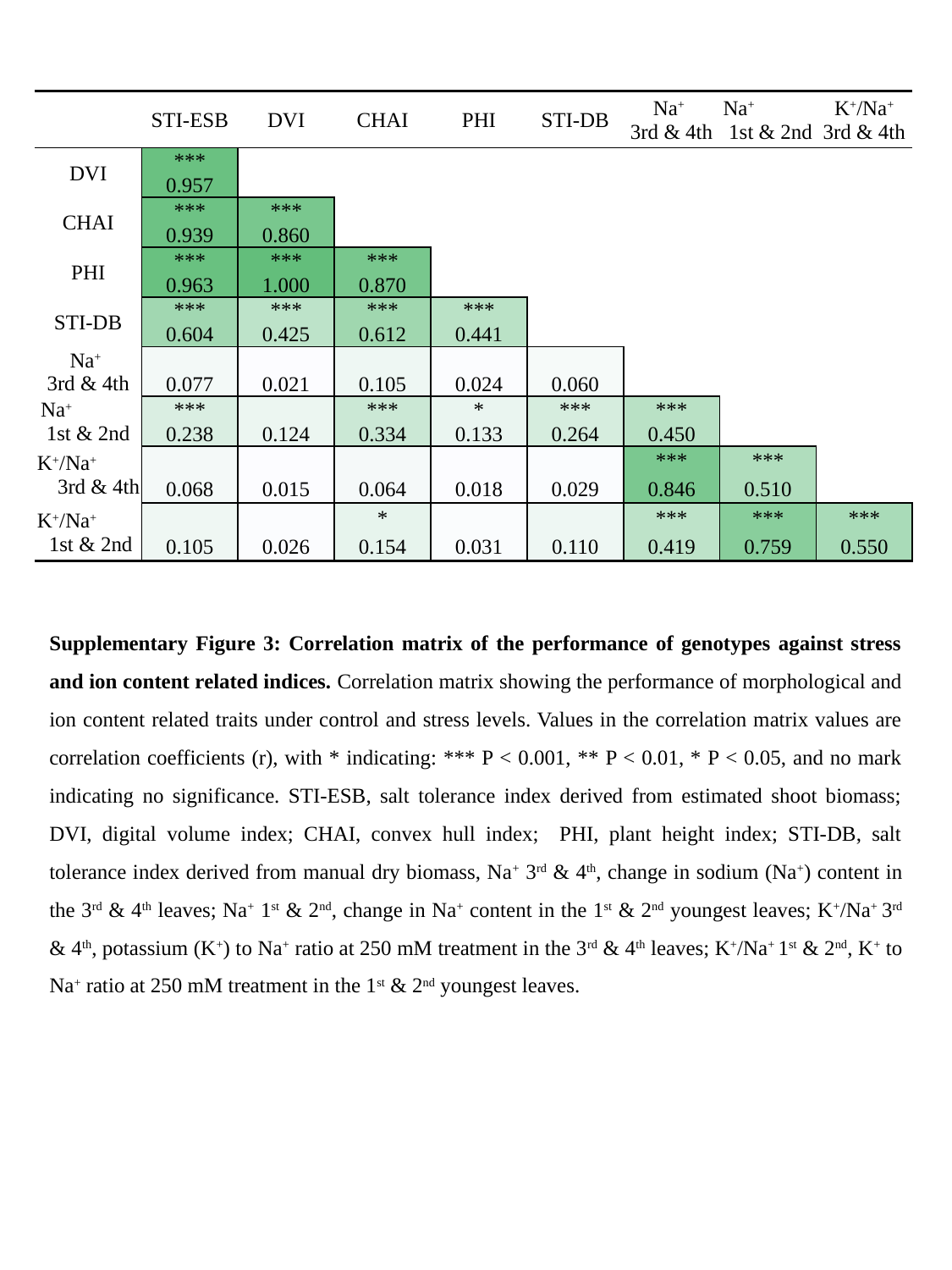

| | STI-ESB | DVI | CHAI | PHI | STI-DB | Na+ 3rd & 4th | Na+ 1st & 2nd | K+/Na+ 3rd & 4th |
| --- | --- | --- | --- | --- | --- | --- | --- | --- |
| DVI | \*\*\* | | | | | | | |
| | 0.957 | | | | | | | |
| CHAI | \*\*\* | \*\*\* | | | | | | |
| | 0.939 | 0.860 | | | | | | |
| PHI | \*\*\* | \*\*\* | \*\*\* | | | | | |
| | 0.963 | 1.000 | 0.870 | | | | | |
| STI-DB | \*\*\* | \*\*\* | \*\*\* | \*\*\* | | | | |
| | 0.604 | 0.425 | 0.612 | 0.441 | | | | |
| Na+ 3rd & 4th | | | | | | | | |
| | 0.077 | 0.021 | 0.105 | 0.024 | 0.060 | | | |
| Na+ 1st & 2nd | \*\*\* | | \*\*\* | \* | \*\*\* | \*\*\* | | |
| | 0.238 | 0.124 | 0.334 | 0.133 | 0.264 | 0.450 | | |
| K+/Na+ 3rd & 4th | | | | | | \*\*\* | \*\*\* | |
| | 0.068 | 0.015 | 0.064 | 0.018 | 0.029 | 0.846 | 0.510 | |
| K+/Na+ 1st & 2nd | | | \* | | | \*\*\* | \*\*\* | \*\*\* |
| | 0.105 | 0.026 | 0.154 | 0.031 | 0.110 | 0.419 | 0.759 | 0.550 |
Supplementary Figure 3: Correlation matrix of the performance of genotypes against stress and ion content related indices. Correlation matrix showing the performance of morphological and ion content related traits under control and stress levels. Values in the correlation matrix values are correlation coefficients (r), with * indicating: *** P < 0.001, ** P < 0.01, * P < 0.05, and no mark indicating no significance. STI-ESB, salt tolerance index derived from estimated shoot biomass; DVI, digital volume index; CHAI, convex hull index; PHI, plant height index; STI-DB, salt tolerance index derived from manual dry biomass, Na+ 3rd & 4th, change in sodium (Na+) content in the 3rd & 4th leaves; Na+ 1st & 2nd, change in Na+ content in the 1st & 2nd youngest leaves; K+/Na+ 3rd & 4th, potassium (K+) to Na+ ratio at 250 mM treatment in the 3rd & 4th leaves; K+/Na+ 1st & 2nd, K+ to Na+ ratio at 250 mM treatment in the 1st & 2nd youngest leaves.
