# Supplementary material for: Digital Phenotyping to Delineate Salinity Response in Safflower Genotypes
Source: Front Plant Sci. 2021 Jun 16;12:662498. doi: 10.3389/fpls.2021.662498 (PMC8242588; doi:10.3389/fpls.2021.662498)
Supplement: Supplementary file 2 [file Data_Sheet_1.docx]

Supplementary Table 1: Safflower genotypes from the Agriculture Victoria diverse safflower population used in glasshouse experiments.

| Genotype | Status | Genotype | Status | Genotype | Status |
| --- | --- | --- | --- | --- | --- |
| AVS-SAFF-4 | Breeder’s Line | AVS-SAFF-80 | Breeder’s Line | AVS-SAFF-138 | Breeder’s Line |
| AVS-SAFF-7 | Breeder’s Line | AVS-SAFF-83 | Breeder’s Line | AVS-SAFF-139 | Breeder’s Line |
| AVS-SAFF-8 | Breeder’s Line | AVS-SAFF-85 | Breeder’s Line | AVS-SAFF-140 | Breeder’s Line |
| AVS-SAFF-11 | Breeder’s Line | AVS-SAFF-86 | Breeder’s Line | AVS-SAFF-144 | Breeder’s Line |
| AVS-SAFF-12 | Breeder’s Line | AVS-SAFF-87 | Breeder’s Line | AVS-SAFF-145 | Breeder’s Line |
| AVS-SAFF-13 | Breeder’s Line | AVS-SAFF-88 | Breeder’s Line | AVS-SAFF-146 | Breeder’s Line |
| AVS-SAFF-14 | Breeder’s Line | AVS-SAFF-89 | Breeder’s Line | AVS-SAFF-148 | Breeder’s Line |
| AVS-SAFF-16 | Breeder’s Line | AVS-SAFF-91 | Breeder’s Line | AVS-SAFF-150 | Breeder’s Line |
| AVS-SAFF-17 | Breeder’s Line | AVS-SAFF-92 | Breeder’s Line | AVS-SAFF-151 | Breeder’s Line |
| AVS-SAFF-18 | Breeder’s Line | AVS-SAFF-94 | Breeder’s Line | AVS-SAFF-152 | Breeder’s Line |
| AVS-SAFF-19 | Breeder’s Line | AVS-SAFF-96 | Breeder’s Line | AVS-SAFF-162 | Landrace |
| AVS-SAFF-21 | Breeder’s Line | AVS-SAFF-97 | Breeder’s Line | AVS-SAFF-163 | Breeder’s Line |
| AVS-SAFF-23 | Breeder’s Line | AVS-SAFF-98 | Breeder’s Line | AVS-SAFF-164 | Breeder’s Line |
| AVS-SAFF-25 | Breeder’s Line | AVS-SAFF-99 | Breeder’s Line | AVS-SAFF-166 | Breeder’s Line |
| AVS-SAFF-26 | Breeder’s Line | AVS-SAFF-100 | Breeder’s Line | AVS-SAFF-169 | Breeder’s Line |
| AVS-SAFF-51 | Breeder’s Line | AVS-SAFF-101 | Breeder’s Line | AVS-SAFF-170 | Breeder’s Line |
| AVS-SAFF-55 | Breeder’s Line | AVS-SAFF-105 | Breeder’s Line | AVS-SAFF-172 | Breeder’s Line |
| AVS-SAFF-56 | Breeder’s Line | AVS-SAFF-112 | Breeder’s Line | AVS-SAFF-173 | Breeder’s Line |
| AVS-SAFF-57 | Breeder’s Line | AVS-SAFF-114 | Breeder’s Line | AVS-SAFF-174 | Breeder’s Line |
| AVS-SAFF-58 | Breeder’s Line | AVS-SAFF-115 | Breeder’s Line | AVS-SAFF-175 | Breeder’s Line |
| AVS-SAFF-62 | Breeder’s Line | AVS-SAFF-116 | Breeder’s Line | AVS-SAFF-177 | Breeder’s Line |
| AVS-SAFF-63 | Breeder’s Line | AVS-SAFF-117 | Breeder’s Line | AVS-SAFF-178 | Breeder’s Line |
| AVS-SAFF-66 | Breeder’s Line | AVS-SAFF-118 | Breeder’s Line | AVS-SAFF-179 | Breeder’s Line |
| AVS-SAFF-67 | Breeder’s Line | AVS-SAFF-121 | Breeder’s Line | AVS-SAFF-181 | Breeder’s Line |
| AVS-SAFF-69 | Breeder’s Line | AVS-SAFF-124 | Breeder’s Line | AVS-SAFF-183 | Breeder’s Line |
| AVS-SAFF-75 | Breeder’s Line | AVS-SAFF-129 | Breeder’s Line | AVS-SAFF-184 | Breeder’s Line |
| AVS-SAFF-77 | Breeder’s Line | AVS-SAFF-130 | Breeder’s Line | AVS-SAFF-187 | Breeder’s Line |
| AVS-SAFF-78 | Breeder’s Line | AVS-SAFF-131 | Breeder’s Line | AVS-SAFF-190 | Breeder’s Line |
| AVS-SAFF-79 | Breeder’s Line | AVS-SAFF-136 | Breeder’s Line | AVS-SAFF-191 | Breeder’s Line |
| AVS-SAFF-192 | Breeder’s Line | AVS-SAFF-239 | Breeder’s Line | AVS-SAFF-281 | Breeder’s Line |
| AVS-SAFF-193 | Breeder’s Line | AVS-SAFF-241 | Breeder’s Line | AVS-SAFF-284 | Breeder’s Line |
| AVS-SAFF-195 | Breeder’s Line | AVS-SAFF-242 | Breeder’s Line | AVS-SAFF-306 | Breeder’s Line |
| AVS-SAFF-202 | Breeder’s Line | AVS-SAFF-243 | Breeder’s Line | AVS-SAFF-307 | Breeder’s Line |
| AVS-SAFF-203 | Breeder’s Line | AVS-SAFF-244 | Breeder’s Line | AVS-SAFF-332 | Breeder’s Line |
| AVS-SAFF-204 | Breeder’s Line | AVS-SAFF-245 | Breeder’s Line | AVS-SAFF-333 | Breeder’s Line |
| AVS-SAFF-206 | Breeder’s Line | AVS-SAFF-246 | Breeder’s Line | AVS-SAFF-334 | Breeder’s Line |
| AVS-SAFF-208 | Breeder’s Line | AVS-SAFF-247 | Breeder’s Line | AVS-SAFF-335 | Breeder’s Line |
| AVS-SAFF-209 | Breeder’s Line | AVS-SAFF-248 | Breeder’s Line | AVS-SAFF-338 | Breeder’s Line |
| AVS-SAFF-210 | Breeder’s Line | AVS-SAFF-249 | Breeder’s Line | AVS-SAFF-341 | Breeder’s Line |
| AVS-SAFF-213 | Breeder’s Line | AVS-SAFF-250 | Breeder’s Line | AVS-SAFF-342 | Breeder’s Line |
| AVS-SAFF-214 | Breeder’s Line | AVS-SAFF-251 | Breeder’s Line | AVS-SAFF-343 | Breeder’s Line |
| AVS-SAFF-216 | Breeder’s Line | AVS-SAFF-252 | Breeder’s Line | AVS-SAFF-345 | Breeder’s Line |
| AVS-SAFF-218 | Breeder’s Line | AVS-SAFF-254 | Breeder’s Line | AVS-SAFF-347 | Breeder’s Line |
| AVS-SAFF-219 | Breeder’s Line | AVS-SAFF-257 | Breeder’s Line | AVS-SAFF-348 | Breeder’s Line |
| AVS-SAFF-220 | Breeder’s Line | AVS-SAFF-260 | Breeder’s Line | AVS-SAFF-349 | Breeder’s Line |
| AVS-SAFF-222 | Breeder’s Line | AVS-SAFF-261 | Breeder’s Line | AVS-SAFF-350 | Breeder’s Line |
| AVS-SAFF-224 | Breeder’s Line | AVS-SAFF-262 | Breeder’s Line | AVS-SAFF-351 | Breeder’s Line |
| AVS-SAFF-225 | Breeder’s Line | AVS-SAFF-263 | Breeder’s Line | AVS-SAFF-352 | Breeder’s Line |
| AVS-SAFF-227 | Breeder’s Line | AVS-SAFF-264 | Breeder’s Line | AVS-SAFF-353 | Breeder’s Line |
| AVS-SAFF-228 | Breeder’s Line | AVS-SAFF-265 | Breeder’s Line | AVS-SAFF-354 | Breeder’s Line |
| AVS-SAFF-230 | Breeder’s Line | AVS-SAFF-267 | Breeder’s Line | AVS-SAFF-355 | Breeder’s Line |
| AVS-SAFF-231 | Breeder’s Line | AVS-SAFF-269 | Breeder’s Line | AVS-SAFF-356 | Breeder’s Line |
| AVS-SAFF-232 | Breeder’s Line | AVS-SAFF-271 | Breeder’s Line | AVS-SAFF-361 | Breeder’s Line |
| AVS-SAFF-234 | Breeder’s Line | AVS-SAFF-277 | Breeder’s Line | AVS-SAFF-379 | Breeder’s Line |
| AVS-SAFF-235 | Breeder’s Line | AVS-SAFF-278 | Breeder’s Line | BRIGGS | Advanced cultivar |
| AVS-SAFF-236 | Breeder’s Line | AVS-SAFF-279 | Breeder’s Line | cv.Hamaya 65 | Advanced cultivar |
| AVS-SAFF-237 | Breeder’s Line | AVS-SAFF-280 | Breeder’s Line | cv.Kino 76 | Advanced cultivar |
| Montola2003 | Advanced cultivar | SIGMA 13 | Advanced cultivar | SIGMA 46 | Advanced cultivar |
| PI 538025 | Advanced cultivar | SIGMA 16 | Advanced cultivar | SIGMA 48 | Advanced cultivar |
| ROYAL | Advanced cultivar | SIGMA 19 | Advanced cultivar | SIGMA 50 | Advanced cultivar |
| S317 | Advanced cultivar | SIGMA 24 | Advanced cultivar | SIRONARIA | Advanced cultivar |
| CW 99-OL | Advanced cultivar | SIGMA 25 | Advanced cultivar | UC 148 | Advanced cultivar |
| FRIO | Advanced cultivar | SIGMA 27 | Advanced cultivar | USB | Advanced cultivar |
| Gila | Advanced cultivar | SIGMA 29 | Advanced cultivar | VARIETY 1 CSIRO ORD RIVER | Advanced cultivar |
| LEED | Advanced cultivar | SIGMA 38 | Advanced cultivar | VARIETY 5 CSIRO ORD RIVER | Advanced cultivar |
| LESEF 174 | Advanced cultivar | SIGMA 42 | Advanced cultivar | GILA 27 PACIFIC SEED U.S.A. | Advanced cultivar |
| SIGMA 11 | Advanced cultivar | SIGMA 45 | Advanced cultivar |  |  |

Supplementary Table 2: Description of salt solution calculations for glasshouse experiments.

| Salt Treatment | Application Days | Salt Concentration per Application (mM) | Volume dispensed per application (mL) | Volume of 1M Stock per application (mL)* | Volume of water for application (mL) |
| --- | --- | --- | --- | --- | --- |
| 0 mM | 2 days | 0 | 150 | 0 | 1000 |
| 125 mM | 2 days | 62.5 | 150 | 562 | 438 |
| 250mM | 3 days | 83.3 | 150 | 749 | 251 |
| 350 mM | 3 days | 116.6 | 157 | 1000 | 0 |

*Calculated using the formula [(SGWC x Salt concentration per application)/ Volume dispensed per application]. Soil gravimetric water content (SGWC) was 1350g in this experiment.
